# Case Report: Extensive Temporal Bone Invasion in a Giant Vestibular Schwannoma

**DOI:** 10.3389/fsurg.2022.759163

**Published:** 2022-05-25

**Authors:** Fang Lyu, Jinlu Gan, Haijun Wang, Hongyang Zhao, Lei Wang, Fangcheng Zhang

**Affiliations:** Department of Neurosurgery, Union Hospital, Tongji Medical College, Huazhong University of Science and Technology, Wuhan, China

**Keywords:** vestibular schwannoma, acoustic neuroma, temporal bone invasion, gamma knife surgery, microscopic surgery, inflammation

## Abstract

**Background:**

Rare giant vestibular schwannomas (GVSs) invade the temporal bone extensively, which carries unique risks for surgery owing to their complicated relationship with adjacent structures, difficult dissection of the temporal bone, and high risk of complications. The underlying mechanism of this invasive behavior remains unknown.

**Case description:**

We report on a 28-year-old woman who presented with typical hearing loss and facial paralysis (House-Brackmann II). Magnetic resonance imaging exhibited a giant mass (∼5.0 cm) in the right cerebellopontine angle (CPA), petrous apex, and middle cranial fossa. Her primary diagnosis was GVS with petrous apex invasion. With the aid of presurgical imaging reconstruction and intraoperative facial nerve monitoring, we adopted a sequential therapeutic strategy, which included microsurgery for the CPA lesion followed by gamma knife radiosurgery (GKRS) for the petrous mass. During follow-up, stable tumor control was achieved with functional preservation of the facial nerve and no other complications. The postoperative immunohistochemical examination demonstrated dramatic intratumoral inflammation, which suggested its potential role in bony erosion. We reviewed the literature of large vestibular schwannoma with a petrous invasion and further discussed its treatment.

**Conclusion:**

Microsurgery remains the top therapeutic strategy for GVS. However, gross total resection with functional preservation of cranial nerves is challenging to achieve once the temporal bone is involved. In this case, we applied a planned and sequential approach of microsurgery and GKRS with a promising outcome, which highlighted this combinational strategy in this rare situation. In addition, pathological examination suggested that intratumoral inflammation might play a role in the bony erosion of GVS. Longer observation and more cases are needed to further investigate its molecular mechanism and treatment plan.

## Introduction

Vestibular schwannoma (VS) is a primary tumor of the 8th cerebral nerve. VSs account for 8% to 10% of intracranial tumors, and their incidence rate dramatically increased from 3 to 34 cases per million annually over just a few decades (1, 2). A typical giant vestibular schwannoma (GVS) arises in the internal auditory canal (IAC) and gradually grows and expands to the cerebellopontine cistern with a diameter larger than 40 mm (3, 4). Almost all GVSs would widen IAC, but they rarely erode the temporal bone aggressively and extend to the middle cranial fossa. Their exact mechanism remains unknown.

Microsurgery and gamma knife radiosurgery (GKRS) are the primary treatment methods for VSs. However, the literature regarding therapeutic plans for a GVS with petrous invasion was limited. To the best of our knowledge, no studies have reported a planned and sequential approach of microsurgery and GKRS for this rare condition. In this study, we reported the case of a young woman with GVS and petrous apex erosion. We adopted a sequential approach of microsurgery and GKRS and achieved satisfactory tumor control. Meanwhile, a pilot exploration of intratumoral inflammation was perform in this patient to investigate its role in bony invasion. We further reviewed the literature of large VSs with petrous invasion and discussed its therapeutic strategy.

## Case Description

**Figure 1:**
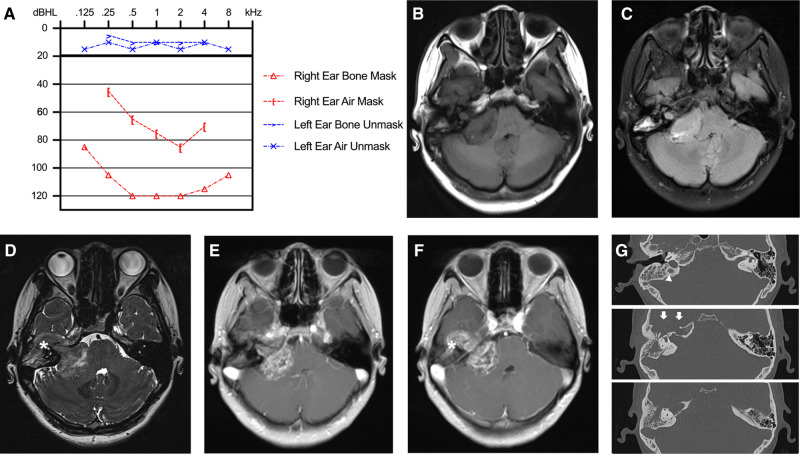
Preoperative hearing and imaging scans. (**A**) Preoperative pure tone threshold audiometry demonstrated profound mixed hearing impairment of the right ear. (**B–F**) MRI scan revealed a mass with a maximal diameter of 5.0 cm at the right cerebellopontine angle with petrous bone invasion. The lesion exhibited low- to iso-intense on T1-weighted images (**B**), iso- to high-intense on T2-FLAIR images (**C**), mixed intense on constructive interference in steady state (CISS) images (**D**), and heterogeneously enhanced after Gd-DTPA administration (**E,F**). (**G**) In the CT scan, we observed an enlargement of the right IAC with significantly bony destruction of the temporal apex (white arrows) and a slightly high ride of the jugular bulb (white triangle).

**Figure 2:**
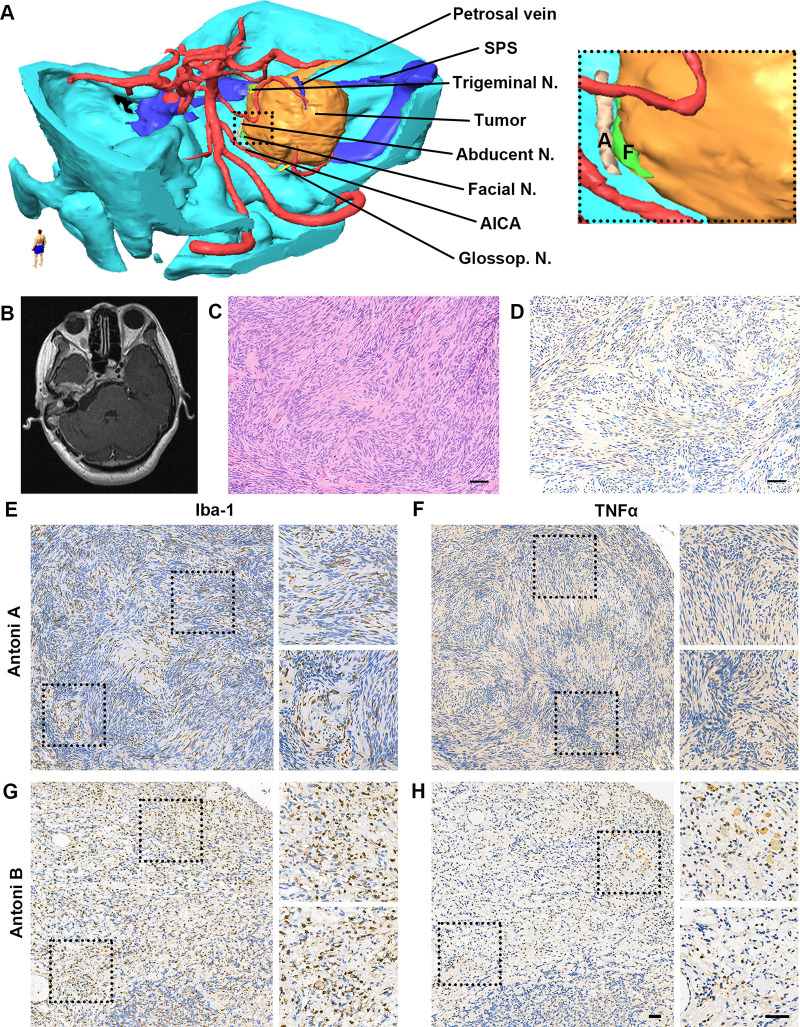
Reconstruction tumor model, imaging scan and pathological examination after surgery. (**A**) Three-dimensional tumor model was reconstructed based on preoperative MRI and CT scan to demonstrate the spatial relationship of the tumor, adjacent nerves, arteries, and veins. (**B**) Postoperative MRI scan exhibited near-total resection of GVS in the posterior cranial fossa. (**C,D**) Pathological examination exhibited typical Verocay bodies in Antoni A region with positive S100 immunoactivity (**C**. H&E staining; **D**. S100b+ cells in IHC. Scale bar, 50 μm). Abbreviations, AICA, anterior inferior cerebellar artery; N., nerve; SPS, superior petrous sinus. (**E-H**). High immunoactivity of Iba-1 and TNFα in the tumor. (**E**) Iba-1 expression in Antoni A region; (**F**) TNFα expression in Antoni A region; (**G**) Iba-1 expression in Antoni B region; (**H**) TNFα expression in Antoni B region. Scale bar, 50 μm. Abbreviations, Iba-1, ionized calcium binding adaptor molecule 1; TNFα, tumor necrosis factor-alpha.

A 28-year-old woman presented with hearing loss for five years, intermittent dizziness and headache for one month, and facial paralysis for eight days (House-Brackmann II). On admission, neurological and physical examination revealed profound mixed hearing impairment of the right ear (116 dBHL; Figure 1A). No other apparent abnormalities were detected in her facial sensory, mastication function, and tone of voice. No systematic disease, family history of neurofibromatosis, or other genetic or bone diseases were reported in her past. Magnetic resonance imaging (MRI) revealed a dumbbell mass with a maximal diameter of approximately 5.0 cm at the right cerebellopontine angle (CPA) and the temporal bone (Figures 1B–F). The lesion compressed the brainstem significantly and exhibited low- to iso-intense on T1-weighted images and iso- to high-intense on T2 fluid-attenuated inversion recovery images and heterogeneously enhanced after gadolinium-diethylenetriamine pentaacetic acid administration (Figures 1B–F). In the computed tomography (CT) scan, we observed an enlargement of the right IAC with significantly bony destruction of the temporal bone (Figure 1G). There was suspicious soft tissue in the mastoid cells, which was later proved to be part of the tumor during the surgery (Figure 1G). Based on MRI and CT scans, BrainLab iPlan Stereotaxy software (version 3.0.6; BrainLab, Munich, Germany) was applied to reconstruct the three-dimensional model to demonstrate the spatial relationship of the tumor, adjacent nerves, arteries, and veins (Figure 2A).

The patient’s primary diagnosis was GVS with the temporal bone invasion, and a sequential approach of microscopic resection and GKRS was applied. Firstly, microscopic resection was performed via a retrosigmoid suboccipital approach under intraoperative cranial nerve monitoring per established protocol (5–7). Intraoperatively, the tumor was yellow-greyish and moderately tough. Through the aid of preoperative three-dimensional reconstruction and intraoperative neurophysiological monitoring of the facial nerve, the facial nerve was identified in the medial side of the tumor, and we observed the proximal cochleovestibular nerve extending into the tumor, which suggested its cochleovestibular origin. After meticulous arachnoid dissection and intra- and extracapsular decompression, the arachnoid plane at the brainstem was preserved, and a tiny tumor carpet was left on the facial nerve, owing to its extremely high adherence to the nerve. Near-total resection was achieved for the tumor in the posterior cranial fossa. The facial nerve was preserved anatomically and functionally, which was confirmed by the motor unit potential in the proximal stimulating of the nerve root after tumor debulking.

After surgery, no noticeable functional deterioration of the facial nerve or other complications were detected. Postoperative MRI confirmed the extent of microsurgical resection (Figure 2B), and histopathological examination demonstrated typical Verocay bodies in the Antoni A region in hematoxylin and eosin staining with S100b+ in immunohistochemistry (Figures 2C,D).

To further investigate its invasive behavior, the expression of inflammatory markers including biomarkers of macrophage and microglia (ionized calcium-binding adaptor molecule-1 [Iba-1]) and proinflammatory cytokine (tumor necrosis factor-α [TNFα]) was evaluated via immunohistochemistry on 4-μm paraffin slides. For the control, a typical large VS patient was randomly selected from the pathological database. Compared with the control subject (data not shown), obvious higher density of Iba-1 positive cells (Figures 2E,G) was observed in both the Antoni A and B regions with higher expression of TNFα (Figures 2F,H), which also exhibited typical morphology of hyperplastic macrophages and microglia.

**Figure 3:**
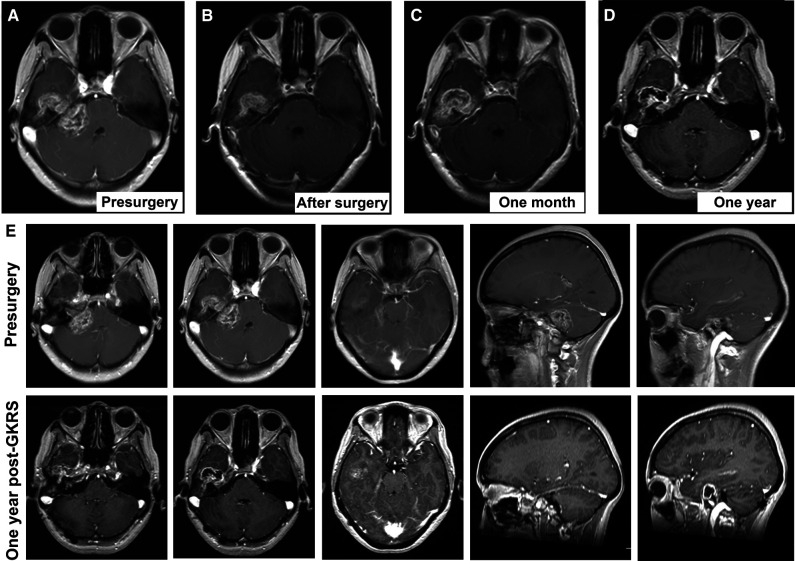
Consecutive follow-up MRI. (**A**) Presurgical scan; (**B**) MRI scan after microsurgery; (**C**) MRI scan one month after gamma knife radiosurgery; (**D**) MRI scan one year after gamma knife radiosurgery. (**E**) the comparison of MRI findings between pre-surgical scan and the scan one year after gamma knife radiosurgery.

Three months after surgery, the patient’s clinical symptoms (dizziness and headache) were relieved, and no residual lesion was observed in the CPA region. GKRS was applied according to plan using the Leksell Model B Gamma Knife (Elekta Instruments, Stockholm, Sweden) for the tumor in the middle cranial fossa and petrous bone. The maximum marginal dose was 14 Gy with a 50% isodose curve. After the GKRS, there were no new symptoms. One month after GKRS, postoperative MRI demonstrated that the petrous part of the tumor had asymptomatic enlargement and degeneration of its central region (Figure 3). One year later, the tumor showed shrinkage of the residual lesion and further cystic degeneration (Figures 3D,E). No functional deterioration of the facial and auditory nerves on the affected side were noticed, and the patient kept good hearing on the healthy ear. During the treatment and follow-up, we kept the communication with this patient and informed her our findings of the condition. In the last clinical visit, the patient felt relieved and satisfied with the outcome.

## Literature Review

Our review involved case reports of GVS with the petrous apex invasion. We searched journal articles in PubMed, Embase, and Web of Science from database inception to May 2021 using the terms “Giant vestibular schwannoma”, “Petrous apex invasion,” “Middle cranial fossa” and “Surgery”. The search was limited to articles in English.

Four eligible reports (seven cases) were identified (Table 1) in the literature. Among the cases reported, five patients were primary VS (8–10) and the rest were recurrent (8, 11). The reports included four men and three women with an average age of 50.7 ± 16.5 years (age range, 27–77 years old). The patients’ main complaints were hearing loss and headache, and the average maximum diameter of the tumors was 42.8 ± 10.6 mm (range, 34–60 mm, from five available cases) (8–11). Microsurgery was the dominant therapeutic strategy. Of the patients, four underwent microscopic resection via a modified retrosigmoid approach, one via a classic transmastoid translabyrinthine approach and the other two via a combinational translabyrinthine and retrosigmoid approach (8–11). However, four patients achieved gross total resection, only one had functional preservation of the facial nerve, and two were clarified with no other complications (8–11).

**Table 1:** Literature review of large VS with temporal bone invasion.

Author	Year	Gender	Age	Max. D.	Treatment	Outcome	Facial N. (H-B)	Follow up
Feghali JG	1995	M	43	35	Transcranial suboccipital translabyrinthine approach	GTR	IV	N.A.
		F	57	34	Transcranial suboccipital translabyrinthine approach	NTR	III	12 months
		F	38	60	Transcranial suboccipital translabyrinthine approach	N.A.	IV	10 months
		F	77	N.A.	Transmastoid translabyrinthine approach	GTR	N.A.	N.A.
Matsumura H	2019	M	62	N.A.	Transmastoid translabyrinthine and retrosigmoid approach	STR	I	N.A.
Park SJ	2015	M	51	40	Translabyrinthine and retrosigmoid approach	GTR	I	18 months
Sato Y	2017	M	27	45	Retrosigmoid intradural suprameatal-inframeatal approach	GTR	IV	N.A.

*Abbreviations: F, Female; M, Male; N, No; N.A., Not Available; Y, Yes; Max. D., Maximum Diameter; H-B, House-Brackmann; mths, months; GTR, Gross Total Resection; STR, Subtotal Resection.*

## Discussion

VSs usually originate from the vestibular ganglion and develop at an insidious rate despite their benign nature in pathology. If left untreated, some VSs could grow more than 4 cm in one dimension (GVS) and compress adjacent cranial nerves, vessels, and the brainstem (3). Typical clinical presentations include hearing loss, tinnitus, vertigo, and facial numbness (12). In extremely rare cases, the GVS invades the petrous apex and extends to the middle cranial fossa. The clinical manifestations of GVS with petrous apex invasion are similar to typical VS. However, mixed hearing loss may be unique in this condition, owing to the invasion of the temporal bone.

Most of the literature regarding GVS was clinical and imaging research. The mechanism of the invasive behavior remains unknown. In a radiological study on the association of IAC widening and tumor characteristics, Takada et al. proposed that the potential mechanism underlying IAC destruction included (1) repeated intratumoral strokes that led to a gradual erosion of the bony wall of IAC; and (2) increased cerebrospinal fluid pressure on the site and solidity of the tumor also contributed the bone erosion (13). As for petrous invasion, Feghali et al. hypothesized that the tumor could invade the petrous bone through the pneumatised tract after the breakthrough of the IAC cortex, during which individual factors, such as temporal bone consistency and thick membranous scar secondary to the primary surgery, also played a role in this process (8).

In the present case, we investigated various factors related to extensive invasion of the temporal bone. However, the patient exhibited no signs of osteoporosis, and her laboratory examinations, including serum levels of calcium, phosphorus, and alkaline phosphatase, were within physiological levels. She also had no history of head trauma, and her preoperative CT scan showed normal pneumatization of the temporal bone. During the surgery, the tumor showed a typical VS pathological appearance. Together with her presurgical imaging, there was no evidence of intratumoral stroke. Her histological findings demonstrated typical benign VS features, and there was no unusual vascularity or any malignant features. Notably, the suspicious soft tissue in the mastoid cells detected in CT scans was confirmed to be part of the tumor during the surgery, which suggested that the lesion eroded the cortex of the IAC and invaded the bone through the pneumatised tract.

Per established study in diseases related to common bony destruction, such as rheumatoid arthritis and bone metastases, systematic and regional inflammation could lead directly to this bony change via promotion, differentiation, and maturation of osteoclast (14). Osteoclasts are formed by differentiation and fusion of precursor cells derived from a monocyte or macrophage lineage whose physiological differentiation requires the tumor necrosis factor superfamily member (15, 16). Although a variety of inflammatory cytokines, such as interleukin-1 and interleukin-6, contribute to osteoclastogenesis, TNFα appears to be the predominant molecule that mediates inflammatory osteolysis through its p55 receptor (15, 17). It has recently been reported that secondary releasing of TNFα played a central role in inflammatory osteoclastogenesis and bony destruction, rather than tumor cells themselves, in patients with invasive pituitary adenomas (18). Moreover, relevant literature demonstrated that the expression level of TNFα in VS increased observably in comparison with healthy vestibular nerve samples (19). Therefore, we hypothesize that a similar inflammatory response may contribute to bony erosion in GVS. Interestingly, high immunoactivity of Iba-1 and TNFα was observed in both the Antoni A and B regions of the sample (Figures 2E–H), which suggested robust activation of macrophage and microglia with increased release of TNFα. The simultaneous presence of inflammation and the bony invasion in this case suggested their potential relationship in this GVS. However, more cases and systematic molecular and cellular experiments are needed to verify this finding.

For neurosurgeons, GVS carries unique risks for microsurgical resection, owing to their large dimensions, deep location, and complicated relationship with adjacent vessels and nerves. GVS invading the petrous apex is extremely rare, making it impractical for resection alone, owing to its high risk of severe complications and treatment failure (20). The limited literature summarised above (Table 1) shows that the primary therapeutic strategy for bone invasive GVS was microsurgery. Due to the mass located in both the CPA and temporal bone, the translabyrinthine and modified retrosigmoid are the most common approaches. However, the preservation rate of facial nerve function and the extent of resection was unsatisfactory, owing to difficult dissection in the temporal bone and prolonged manipulation of the nerves. Recently, Chiang et al. retrospectively reviewed 104 patients with large cerebellopontine angle tumor >3 cm treated by the microsurgery via a translabyrinthine approach. They observed that the extent of tumor removal in the internal acoustic meatus was associated with poor postoperative facial function, and larger tumor size with younger age was indicators for radiosurgery (21). Another surgical approach applied is transotic approach developed by Fisch et al. in 1988 (22). In comparison with translabyrinthine approach, the transotic approach provides a better exposure to the anterior cerebellopontine angle, which favors the preservation of the facial nerve and radical tumor removal without little brain retraction (23). Previous studies recommended this approach for the tumor around or less than 3 cm to optimize the clinical prognosis. Whereas, Xia et al. successfully performed the transotic approach in the GVS (4.0∼5.0 cm) with low rat of complications (e.g., cerebral spinal fluid leakage, intracranial hemorrhage, vertigo, etc.) (23). It would be valuable to evaluate this approach in the future cases.

GKRS is an alternative treatment option for vestibular schwannomas (24). In previous clinical studies, GKRS has been considered particularly suitable for small- and medium-sized lesions (maximum diameter <30 mm) with satisfactory tumor control with low risk of nerve dysfunction (25–28). However, single dose GKRS is not the primary choice for large and giant VS, owing to the high rate of treatment failure (especially tumors >15 cm^3^), transient tumor expansion after surgery, large dose radiation, and secondary intratumoral and peritumoral edema.(25, 28–30). Besides the single dose protocol, there is emerging evidence suggesting that fractioned GKRS is a promising strategy for treating large intracranial lesions (31, 32). Theoretically, fractioned treatment plans can reduce the adverse effect on the surrounding tissue in each session and improve the overall outcome via increasing the total dose (31). In VSs, McTyre et al. prescribed a dose of 20 Gy in four fractions and achieved a stable tumor control with hearing preservation in four VSs patients (32). However, the clinical application of fractionated GKRS in large VSs is rare and the treatment protocol need to be further investigated. Another key proceeding of the radiotherapy in recent years is the introduction of chemical radiosensitizers and radioenhancers in the VS treatment (33, 34). In the experimental VSs associated with neurofibromatosis type II (NF2), targeting cMET pathways and blockage of VEGF signalling was reported to augment radiation response without adverse effect on hearing (34, 35). In future studies, it would be valuable for clinicians and researchers to evaluate their safety and efficacy in GKRS.

In this study, we applied a planned and sequential approach of microsurgery and GKRS, which may serve as a better option and overcome the shortcomings of each individual treatment. It has been reported that planned subtotal resection followed by GKRS could achieve good tumor growth control, nerve function preservation, and a lower risk of complications in large VS (36, 37). However, there are no published reports on its application in GVS with the temporal bone invasion. After discussing three potential treatments with the patient, we reached a consensus and adopted this sequential approach. Our logic was to radically reduce the tumor volume (major in the CPA region) to a dimension suitable for GKRS and then perform GKRS for the residual located in the petrous apex. To protect the facial nerve, both preoperative imaging reconstruction and intraoperative cranial nerve monitor were applied. During the patient’s last clinical visit, the facial nerve’s function remained similar to its presurgical status (House-Brackmann II). No cerebral spinal fluid leakage, cerebellar atrophy, or dysfunction of other cranial nerves was detected. In the follow-up MRI, the tumor was near-total resected in the CPA region, and the petrous part had a transient expansion in the first month after GKRS and started to shrink in the ninth month. Based on the limited information distilled in this case and our literature review, we suggest that this sequential therapeutic strategy might be safe and curative in the bone invasive GVS.

However, there are several limitations in this case. First of all, there is only one patient in this report, and more cases are needed to verify this therapeutic strategy and explore the molecular mechanism underlying invasive behavior of GVS. Besides, the time of clinical follow-up post GKRS was limited. As reported previously, GKRS in a higher dose (18 Gy) could lead to high occurrence of late complications, e.g., osteoradionecrosis, fibrosis, trismus, and etc. (38) Thus, longer observation is needed to further evaluate the complications risk after GKRS.

## Conclusions

GVS with extensive temporal invasion is rare, and microsurgery remains the primary therapeutic strategy. However, gross total resection with functional preservation of cranial nerves is not easy to achieve. In this case, we applied a planned and sequential approach of microsurgery and GKRS with a satisfactory outcome, which highlighted this combinational strategy in this rare situation. In addition, pathological examination suggested that intratumoral inflammation might play a role in the bony erosion of GVS. Longer observation periods and more cases are needed to further investigate its molecular mechanism and treatment plan.

## Data Availability

The original contributions presented in the study are included in the article/Supplementary Material, further inquiries can be directed to the corresponding author/s.
